# Comparison of Outcomes in Early- and Late-Tracheostomised Head Injury Patients

**DOI:** 10.7759/cureus.112629

**Published:** 2026-07-13

**Authors:** Akansha Singh, Priyanka Verma, Aditya Gargava

**Affiliations:** 1 Department of Ear, Nose, and Throat (ENT), Atal Bihari Vajpayee Government Medical College, Vidisha, IND

**Keywords:** glasgow coma scale, intensive care unit, mechanical ventilation, tracheostomy group, ventilator-associated pneumonia

## Abstract

Background: Early tracheostomy (ET) has been associated with improved airway stability and reduced complications in mechanically ventilated patients. Its optimal timing in severe traumatic brain injury (TBI) remains debated, particularly because outcomes may be influenced by baseline neurological severity, associated injuries, and clinical selection for tracheostomy timing.

Objective: The study aimed to compare unadjusted clinical outcomes between ET (<4 days) and late tracheostomy (LT) (>4 days) in patients with severe head injury and a Glasgow Coma Scale (GCS) score <8.

Methods: This retrospective comparative study included 120 patients with severe TBI managed over 18 months. Patients were classified into ET (n=60) and LT (n=60) groups based on the timing of the procedure. Outcomes analysed included duration of mechanical ventilation (MV), intensive care unit (ICU) stay, hospital stay, time to ward transfer, ventilator-associated pneumonia (VAP), and final clinical outcome. The analysis was unadjusted, and multivariable regression was not performed because complete covariate data for injury severity, associated injuries, sedation exposure, respiratory status, and weaning-related factors were not uniformly available in the retrospective records. Statistical significance was set at p<0.05.

Results: Mean age was 43.08 years in the ET group and 39.27 years in the LT group. In the unadjusted analysis, mean DMV was significantly shorter in the ET group (8.45 ± 4.09 days) compared with the LT group (13.73 ± 4.47 days; p<0.05). ICU stay was significantly shorter in the ET group (11.93 ± 5.24 days) than in the LT group (19.00 ± 7.02 days; p<0.05). VAP incidence was numerically lower in the ET group, with five patients (8.3%), than in the LT group, with 12 patients (20.0%), although this difference was not statistically significant. Clinical improvement was significantly higher in the ET group, with 49 patients (81.7%), compared with the LT group, with 27 patients (45.0%). The ET group had higher day 1 and day 3 GCS values than the LT group, indicating baseline neurological imbalance between groups.

Conclusion: In this retrospective, unadjusted cohort, ET in patients with severe TBI was associated with shorter duration of MV, reduced ICU stay, earlier ward transfer, and better short-term clinical outcomes compared with LT. These findings should be interpreted cautiously because the groups were not fully comparable in neurological severity, no prospective sample size calculation was performed, and multivariable adjustment for confounding was not possible. The results represent associations rather than evidence that ET independently caused improved outcomes.

## Introduction

Traumatic brain injury (TBI) is an important cause of morbidity and mortality worldwide and commonly requires airway protection and ventilatory support in the acute stage [1]. Patients with severe TBI often need intubation to ensure airway patency, prevent hypoxia, and allow controlled ventilation because of impaired consciousness and impaired protective reflexes [2]. In this setting, tracheostomy represents an important airway management strategy, especially in patients who are expected to require prolonged mechanical ventilation (MV) or those with difficulty in airway toileting and secretion clearance [3].

Tracheostomy is usually performed in intensive care units (ICUs) for several reasons, such as failure to wean off MV, absent protective airway reflexes, low respiratory drive, and difficulty managing secretions [2]. In patients with neurological injury, other contributing factors to the decision include low Glasgow Coma Scale (GCS), poor cough reflex, and persistent altered sensorium [4]. Despite its widespread use, there is continued controversy about when tracheostomy should be performed in patients with severe head injury [1].

Historically, the definition of early tracheostomy (ET) has been variable [5]. In the 1980s, tracheostomy was defined as early if performed before 21 days of intubation [2]. With improvements in critical care protocols and increasing focus on reducing ventilator days and ICU burden, clinical practice has changed [6]. Contemporary studies often define ET as tracheostomy performed within 2-10 days of MV, although variability remains [2]. Some investigators define ET as tracheostomy performed within three days, with procedures performed after day 4 classified as late tracheostomy (LT) [3]. Such inconsistency adds heterogeneity to reported outcomes and complicates the interpretation of available evidence [1].

Early initiation of physical therapy in trauma patients, including those with TBI, has been associated with improved clinical recovery and may contribute to earlier functional progression during hospitalisation [7].

ET has been suggested to reduce airway resistance, improve pulmonary toileting and mobilisation, reduce sedation requirements, and shorten MV duration and ICU stay [8]. These possible benefits make ET an attractive approach in the management of neurotrauma, in which prolonged ventilatory support is common [9].

Evidence concerning ET remains inconsistent [1]. Some investigations report decreased duration of MV and shorter ICU stay with early intervention; however, others show limited benefit or identify risks, such as procedure-related complications and increased mortality in selected subgroups [8]. Shorter MV duration has been found with ET, although higher in-hospital mortality has also been reported in some analyses, raising concerns about patient selection and confounding [8]. Similarly, findings from neurosurgical ICU studies differ according to injury severity, associated trauma, and institutional protocol [10].

Reduced MV duration and ICU stay in patients with TBI undergoing ET have been documented [4,8,9]. Additional reports describe fewer ventilator days and favourable ICU outcomes among neurotrauma patients undergoing ET [9]. Improved clinical outcomes in neurosurgical populations have also been associated with earlier tracheostomy timing [11]. However, the degree of benefit may vary across patient groups, and standardised predictors for guiding optimal timing remain limited [12].

The debate is also influenced by ventilator-associated pneumonia (VAP), which is a major nosocomial infection in mechanically ventilated patients [13]. The timing of tracheostomy has clinical relevance because prolonged endotracheal intubation is associated with complications, including laryngeal trauma, increased sedation requirements, discomfort, impaired oral hygiene, and increased risk of VAP [13]. VAP is associated with prolonged ventilator duration, longer ICU stay, higher medical expenses, and increased morbidity [14]. ET may help decrease the incidence of VAP by shortening the duration of translaryngeal intubation and improving airway toileting [15]. Nevertheless, the association between tracheostomy timing and VAP is not always consistent, with some studies showing lower rates and others showing no significant difference [1,2].

In critically ill adult populations other than neurotrauma, ET is linked to reduced ICU duration and earlier liberation from ventilation [11]. Improved outcomes have been reported in patients requiring prolonged MV when tracheostomy was performed earlier [12]. Comparative studies of early versus late percutaneous tracheotomy in critically ill patients have shown differences in ventilatory time and ICU length [11]. Differences in neurological recovery and impaired airway reflexes in severe head injury populations may limit direct generalisation of these findings [13].

Objectives of the study

The primary objective of this study was to compare the unadjusted duration of MV between patients with severe TBI and a GCS score <8 who underwent ET, defined as tracheostomy performed within four days of injury, and those who underwent LT, defined as tracheostomy performed after four days of injury. The secondary objectives were to compare unadjusted differences in ICU stay, total hospital stay, time to transfer to the general ward, incidence of VAP, and final clinical outcomes between the early and late tracheostomy groups. Because this was a retrospective observational study without severity matching or multivariable adjustment, these comparisons were intended to identify associations rather than independent causal effects of tracheostomy timing.

## Materials and methods

Study design and study setting

This retrospective comparative observational study was conducted in the Trauma Centre and Neurosurgery Ward of Jayarogya (J.A.) Group of Hospitals, Gwalior, India. The study evaluated the clinical outcomes of patients with TBI who underwent early or late tracheostomy. Data were collected by reviewing hospital records of eligible patients admitted over 18 months from July 2022 to December 2023. The reviewed records included admission notes, ICU charts, neurological assessment records, ventilator records, operative notes, chest radiography reports, laboratory records, and discharge summaries. Demographic data, GCS scores, timing of tracheostomy, duration of MV, ICU stay, hospital stay, VAP status, indication for tracheostomy, and final clinical outcomes were extracted using a predefined data collection format. During admission, all patients received care according to institutional neurocritical care protocols, including institutional MV and weaning guidelines.

Ethical approval

The Institutional Ethics Committee of Gajra Raja Medical College, Gwalior, Madhya Pradesh, provided ethical approval before initiation of the study (approval no: 05/IEC-GRMC/2022, dated 01.09.2022). The study was conducted in accordance with the principles of the Declaration of Helsinki and institutional ethical requirements.

Study population and grouping

A total of 120 patients with severe TBI and a GCS score <8 who required ventilatory care were included in the study. Patients were classified into two cohorts according to the timing of tracheostomy. Patients who underwent tracheostomy within four days of head injury were included in the ET group (n=60), whereas those who underwent tracheostomy after four days of head injury were included in the LT group (n=60). All included patients met the predefined criterion for severe TBI, defined by a GCS score <8 and requirement for ventilatory support. The decision regarding tracheostomy timing was made by the treating neurosurgery, intensive care, and ear, nose, and throat (ENT) teams according to routine clinical judgment, including airway protection, secretion burden, anticipated ventilatory requirement, respiratory failure, and expected difficulty in extubation. Because the allocation was not randomised, the two groups cannot be assumed to be fully comparable in terms of baseline TBI severity. Available neurological severity data were therefore reviewed using day 1 and day 3 GCS values, and this potential baseline imbalance was considered during interpretation of outcomes. Because this was a retrospective observational study, tracheostomy timing reflected institutional clinical practice and the observed differences between groups should be interpreted as associations rather than direct causal effects of ET.

Eligibility criteria

Inclusion Criteria

The study included patients aged 14 to 70 years with a clear history of head injury, severe TBI, a GCS score <8, and a requirement for ventilatory care. The lower age limit was selected to exclude pediatric patients because of differences in airway anatomy, ventilatory management, and physiological responses. The upper age limit was used to reduce the influence of advanced age, multiple comorbidities, and age-related physiological decline, which may independently affect outcomes such as duration of MV and ICU stay. Enrollment was restricted to patients with severe brain injury requiring MV to maintain a relatively homogeneous study population in which the timing of tracheostomy would be clinically meaningful.

Exclusion Criteria

Patients younger than 14 years were excluded because of differences in pediatric airway management and critical care procedures. Patients with severe chest or abdominal injuries were excluded to minimise confounding, as such injuries could independently prolong MV and ICU stay. Patients with primary respiratory failure unrelated to head injury were also excluded to ensure that ventilatory dependence was mainly attributable to neurological impairment rather than underlying pulmonary pathology.

Clinical assessment and investigations

All patients underwent detailed clinical history-taking and examination. Neurological status was assessed using the GCS. Baseline investigations included complete blood count and chest radiography, which were repeated after seven days or earlier when clinically indicated. Patients were intubated if their GCS score was less than 8 and were managed with MV. Table 1 presents the components of the GCS for neurological assessment, including eye-opening, verbal, and motor responses with their corresponding scores and interpretation of total score ranges.

**Table 1:** Glasgow Coma Scale Adapted from Teasdale and Jennett’s original Glasgow Coma Scale description [14]. The table has been reformatted for clarity and was not directly reproduced from the original publication; therefore, publisher permission was not required.

Domain	Clinical response	Points
Eye opening	Opens eyes spontaneously	4
	Opens eyes to voice	3
	Opens eyes to painful stimulus	2
	No eye opening	1
Verbal response	Oriented and appropriate	5
	Confused conversation	4
	Inappropriate words	3
	Incomprehensible sounds	2
	No verbal response	1
Motor response	Follows commands	6
	Localises painful stimulus	5
	Withdraws from pain	4
	Abnormal flexion response	3
	Abnormal extension response	2
	No motor response	1
Interpretation of total score	Maximum score	15
	Severe coma	≤8
	Deep coma	3

Tracheostomy procedure

During tracheostomy, the patient was placed in a supine position, and a sandbag was positioned beneath the shoulders to facilitate neck extension. All patients had severe TBI with a GCS score <8 and were already intubated before tracheostomy. The procedure was performed with appropriate anaesthetic and ventilatory support, including intravenous sedation, analgesia, and/or general anaesthetic agents, with or without muscle relaxation, as clinically indicated according to the patient’s condition and institutional anaesthetic practice. Local infiltration at the operative site was used only as an adjunct for surgical-site anaesthesia and hemostasis. The procedure was not performed under local anaesthesia alone. All patients in this study underwent open surgical tracheostomy; percutaneous tracheostomy was not performed in any included patient.

The operative site was identified using Jackson’s safety triangle. A vertical incision was made approximately 2 cm above the sternal notch, beginning at the inferior border of the cricoid cartilage. The deep cervical fascia was exposed, and the strap muscles were dissected and retracted laterally. After identification of the thyroid isthmus, it was either divided or retracted superiorly. The trachea was confirmed in the midline, and a window was created between the second and third tracheal rings. An appropriately sized cuffed, low-pressure, high-volume tracheostomy tube (Sterimed Surgical India Pvt. Ltd., Delhi, India) was inserted. The cuff was inflated, hemostasis was achieved, and the wound was closed in layers.

Correct tracheostomy tube placement was confirmed in all patients using bilateral chest expansion, auscultation of bilateral air entry, ventilator parameters, and oxygen saturation. End-tidal carbon dioxide monitoring was used for confirmation when capnography was available and recorded in the ICU or operation theatre documentation. Because this was a retrospective record-based study, uniform end-tidal carbon dioxide tracing was not documented in all operative or ICU records. This represents a limitation of retrospective procedural documentation and variable recorded availability of capnography data, rather than absence of clinical confirmation of tube placement. Perioperative complications, including haemorrhage, sudden desaturation, tube displacement, and false passage, were monitored.

Post-procedure care

Postoperative care included cuff deflation for five minutes every hour during the first four hours and every fourth hour during the next 24 hours. Adequate humidification was maintained. Suctioning was performed intermittently, with each suctioning episode lasting 30-60 seconds. Suction pressure was maintained between 8 and 15 mmHg, and the suction catheter (Rudraksh Pharma & Surgical Pvt. Ltd., Ahmedabad, India) was not advanced beyond 15 cm.

Diagnosis of VAP

VAP was defined as pneumonia that developed at least 48 hours after initiation of invasive MV in patients who had no clinical evidence of pneumonia before or during intubation. Diagnosis was based on the Clinical Pulmonary Infection Score (CPIS) [15], which includes clinical and radiological parameters such as temperature, leukocyte count, pulmonary secretions, oxygenation status, and chest radiography findings. Table 2 lists the CPIS criteria used for diagnosing VAP.

**Table 2:** Clinical Pulmonary Infection Score criteria Adapted from the Clinical Pulmonary Infection Score described by Pugin et al. [15]. The table has been reformatted for clarity and was not directly reproduced from the original publication; therefore, publisher permission was not required. WBC: White blood cell, PaO_2_: Partial pressure of arterial oxygen, FiO_2_: Fraction of inspired oxygen, ARDS: Acute respiratory distress syndrome

Criterion	Values	Score
Temperature (°C)	36-38.4	0
	38.5-39.8	1
	<36 or >39	2
WBC count (cells/mm³)	4,000-11,000	0
	<4,000 or >11,000	1
	Bands >500	2
Pulmonary secretions	Absence of pulmonary secretions	0
	Presence of non-infectious pulmonary secretions	1
Oxygenation (PaO₂/FiO₂)	>240 or presence of ARDS signs	0
Chest X-ray	No infiltrate	0
	Diffuse infiltrate	1
	Localised infiltrate	2

Weaning criteria and patient transfer

Patients were reassessed clinically for readiness to wean from MV. Readiness for weaning was defined by stable arterial blood gas parameters, partial pressure of arterial carbon dioxide (PaCO_2_) <60 mmHg, absence of respiratory distress, hemodynamic stability, presence of a gag reflex, and ability to expectorate secretions. After successful weaning, patients were transferred to the general ward.

Outcome measures

The primary outcome was duration of MV. This outcome was interpreted in relation to available baseline clinical severity indicators because ventilatory duration in patients with TBI may be influenced not only by tracheostomy timing but also by neurological severity, associated injuries, respiratory status, secretion burden, hemodynamic stability, and institutional weaning practices. Patients with severe chest or abdominal injuries and primary respiratory failure unrelated to head injury were excluded to reduce major non-neurological confounding; however, residual confounding from TBI severity and other associated injuries could not be fully eliminated in this retrospective design. Secondary outcomes included length of ICU stay, total hospital stay, time from tracheostomy to transfer to the general ward, incidence of VAP, and final clinical outcome.

Statistical analysis

Data were analysed using IBM SPSS Statistics software, version 20.0 (IBM Corp., Armonk, USA). Continuous variables were expressed as mean ± standard deviation (SD). Because complete raw data were not uniformly available for all continuous variables in the retrospective records, robust normality assessment could not be confirmed for every variable. Continuous outcomes were reported as mean ± SD and compared using Student’s t-test according to the original institutional analysis. This approach may be less appropriate for skewed variables and has therefore been acknowledged as a statistical limitation.

The chi-square test was used for categorical variables. A p-value of less than 0.05 was considered statistically significant. No a priori sample size calculation or power analysis was performed because this was a retrospective census-based study that included all eligible patients admitted during the defined 18-month study period. The sample size was therefore determined by the number of eligible records available during the study period rather than by prospective sample-size estimation. This limits the precision of the estimates and has been acknowledged as a methodological limitation.

Baseline comparability between the early and late tracheostomy groups was assessed using available demographic and neurological variables, including age, sex distribution, and GCS values. Patients with severe chest or abdominal injuries and primary respiratory failure unrelated to head injury were excluded to minimise major confounding from non-neurological causes of prolonged ventilation. Potential predictors of duration of MV included neurological severity, associated injuries, respiratory complications, secretion burden, sedation exposure, hemodynamic instability, VAP, and institutional weaning decisions. Logistic regression or multivariable regression analysis was not performed because complete data for these potential covariates were not consistently available in the retrospective records. Therefore, the analysis remains unadjusted and cannot establish whether tracheostomy timing independently predicted duration of MV. Accordingly, the reported comparisons should be interpreted only as unadjusted associations and not as evidence of an independent or causal effect of ET on MV duration or other clinical outcomes.

## Results

Demographic characteristics

**Figure 1:**
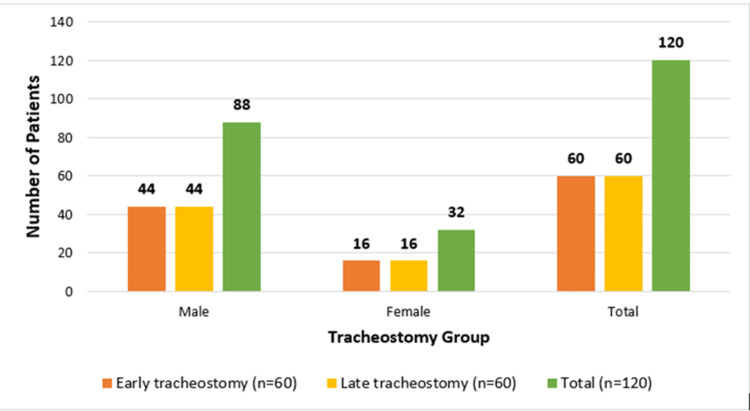
Gender distribution in early and late tracheostomy groups

A total of 120 patients with severe TBI were divided into the ET group (n=60) and the LT group (n=60) based on tracheostomy timing. The ET group's average age was 43.08 ± 13.92 years, whereas the LT group's average age was 39.27 ± 13.49 years, with no statistically significant difference between the groups. Male patients constituted the majority of the study population (73.3%), whereas female patients accounted for 26.7%. Gender distribution was the same in both groups, with 44 male patients and 16 female patients in each group (χ²=0.000, p=1.00). The gender distribution in the early and late tracheostomy groups is depicted in Figure 1, with the same percentages of male and female patients in each group.

GCS comparison

It was observed that the ET group had a significantly higher mean GCS on day 1 (7.62 ± 1.18) than the LT group (6.67 ± 1.14). Mean GCS on day 3 was also significantly higher in the ET group (7.77 ± 3.33) than in the LT group (6.45 ± 1.79). The differences between the groups were statistically significant on both days (p<0.05).

Timing of tracheostomy

The LT group had a significantly later mean timing of tracheostomy (9.50 ± 3.13 days) compared with the ET group (3.37 ± 2.12 days), with a t-value of 12.57 and p<0.05.

MV duration (weaning days)

The mean MV duration (weaning days) was significantly shorter in the ET group (8.45 ± 4.09 days) compared with the LT group (13.73 ± 4.47 days), with a t-value of -6.75 and p<0.05. This comparison was unadjusted and should be interpreted as an association rather than evidence that ET independently reduced MV duration.

ICU stay, transfer to ward, and hospital discharge

In the unadjusted comparison, the mean time from tracheostomy to ward transfer was significantly shorter in the ET group (11.75 ± 5.18 days) compared with the LT group (17.35 ± 5.19 days) (t-value = -5.92, p<0.05). The mean days to discharge were also significantly fewer in the ET group (17.25 ± 5.88 days) than in the LT group (22.52 ± 5.56 days), with a t-value of -5.04 and p<0.05.

The mean total hospital stay was 18.63 ± 14.18 days in the ET group and 23.27 ± 6.40 days in the LT group, and a statistically significant difference was observed (t-value = -2.31, p=0.023). The mean ICU stay was significantly shorter in the ET group (11.93 ± 5.24 days) than in the LT group (19.00 ± 7.02 days), with a t-value of -6.25 and p<0.05. These findings represent unadjusted group comparisons and may have been influenced by baseline neurological severity, associated clinical factors, and institutional weaning or transfer decisions. The Student’s t-test results for continuous variables are summarised in Table 3.

**Table 3:** Comparison of continuous variables between early and late tracheostomy groups ET: Early tracheostomy; LT: Late tracheostomy; ICU: Intensive care unit; SD: Standard deviation

Variable	ET group, mean ± SD	LT group, mean ± SD	t-value	p-value
Timing of tracheostomy, days	3.37 ± 2.12	9.50 ± 3.13	12.57	<0.05
Duration of mechanical ventilation, days	8.45 ± 4.09	13.73 ± 4.47	-6.75	<0.05
Time from tracheostomy to ward transfer, days	11.75 ± 5.18	17.35 ± 5.19	-5.92	<0.05
Days to discharge	17.25 ± 5.88	22.52 ± 5.56	-5.04	<0.05
Total hospital stay, days	18.63 ± 14.18	23.27 ± 6.40	-2.31	0.023
ICU stay, days	11.93 ± 5.24	19.00 ± 7.02	-6.25	<0.05

Indications for tracheostomy

The distribution of indications for tracheostomy was significantly different between the two groups. Prolonged ventilation was the most common indication in the LT group (60.0%), whereas respiratory failure was more common in the ET group (38.3%). Tracheobronchial toileting was documented in 26.7% of patients in the ET group and 21.7% of patients in the LT group. The difference in the distribution of indications between the groups was statistically significant overall (χ² = 8.493, p=0.014), as shown in Table 4.

**Table 4:** Reasons for tracheostomy in early and late tracheostomy groups ET: Early tracheostomy, LT: Late tracheostomy

Reason for tracheostomy	ET group (n=60)	LT group (n=60)	Total (n=120)	Chi-square	p-value
Prolonged ventilation	21 (35.0%)	36 (60.0%)	57	8.493	0.014
Respiratory failure	23 (38.3%)	11 (18.3%)	34		
Tracheobronchial toileting	16 (26.7%)	13 (21.7%)	29		
Total	60 (100%)	60 (100%)	120		

VAP

In the ET group, VAP was observed in five patients (8.3%), whereas in the LT group, it was observed in 12 patients (20.0%). The difference was not statistically significant (χ² = 3.358, p=0.067). Table 5 provides a full comparison between the two groups.

**Table 5:** Comparison of ventilator-associated pneumonia between early and late tracheostomy groups VAP: Ventilator-associated pneumonia, ET: Early tracheostomy, LT: Late tracheostomy

VAP complication	ET group (n=60)	LT group (n=60)	Total (n=120)	Chi-square	p-value
No	55 (91.7%)	48 (80.0%)	103	3.358	0.067
Yes	5 (8.3%)	12 (20.0%)	17		
Total	60 (100%)	60 (100%)	120		

Final clinical outcome

A statistically significant difference was found in final clinical outcomes between the ET and LT groups (χ² = 23.529, p<0.05). Improvement was observed in 49 patients (81.7%) in the ET group compared with 27 patients (45.0%) in the LT group. Complications were more common in the LT group, with 15 patients (25.0%), than in the ET group, with two patients (3.3%). This was an unadjusted categorical comparison, and the observed difference in clinical outcomes may have been influenced by baseline neurological imbalance, injury severity, associated clinical factors, and treatment-related decisions. Therefore, these results should be interpreted as an association rather than evidence that ET independently improved final clinical outcome. The distribution of outcomes is summarised in Table 6.

**Table 6:** Final clinical outcome in early and late tracheostomy groups ET: Early tracheostomy, LT: Late tracheostomy

Outcome	ET group (n=60)	LT group (n=60)	Total (n=120)	Chi-square	p-value
Complications	2 (3.3%)	15 (25.0%)	17	23.529	<0.05
Expired	7 (11.7%)	6 (10.0%)	13		
Improved	49 (81.7%)	27 (45.0%)	76		
Improving	2 (3.3%)	12 (20.0%)	14		
Total	60 (100%)	60 (100%)	120		

In the unadjusted analysis, final clinical outcomes differed significantly between groups, with the ET group showing a higher proportion of improved patients; however, this finding should be interpreted cautiously because baseline neurological imbalance and other unmeasured clinical factors may have influenced the outcome.

## Discussion

Two demographically similar groups participated in the current investigation. The ET group had a mean age of 43.08 ± 13.92 years, whereas the LT group had a mean age of 39.27 ± 13.49 years, with no statistically significant difference between the groups. Male patients accounted for 73.3% of the total sample, suggesting male predominance among patients with severe TBI requiring airway intervention. Similar demographic findings have been reported previously, with 70% of patients being male and a mean age of 46.5 years [5]. Another study reported a similar mean age of 41 years among critically ill patients undergoing tracheostomy [6]. These findings are consistent with the known predominance of severe head injury among male patients.

Neurological assessment using the GCS showed statistically significant differences between the two groups. On day 1, the mean GCS was higher in the ET group (7.62 ± 1.18) than in the LT group (6.67 ± 1.14). By day 3, the ET group showed a mean GCS of 7.77 ± 3.33, whereas the LT group showed a lower mean GCS of 6.45 ± 1.79. These differences indicate that baseline neurological status was not identical between the groups and may have influenced the observed outcomes. Although all patients had severe TBI with a GCS score <8, the higher day 1 and day 3 GCS values in the ET group suggest that the early and late tracheostomy groups were not fully comparable in terms of available neurological severity indicators. This baseline imbalance represents an important potential confounder, as patients with relatively better neurological status may have had shorter ventilatory requirements, shorter ICU stay, and better clinical recovery independent of tracheostomy timing. Although early airway stabilisation may support respiratory management and facilitate clinical care, the present retrospective design does not allow a causal conclusion that ET directly improved neurological status or independently produced better clinical outcomes. The timing of tracheostomy also differed substantially between the ET and LT groups, with a mean timing of 3.37 ± 2.12 days and 9.50 ± 3.13 days, respectively. Indications for tracheostomy included prolonged ventilation, respiratory failure, and tracheobronchial toileting, with prolonged ventilation more common in the LT group. This variation reflects clinical decision-making based on expected ventilatory duration, airway protection, and secretion management. Therefore, the observed outcome differences should be interpreted as associations within a clinically selected retrospective cohort rather than as definitive evidence of causality.

Patients who underwent ET had a significantly shorter duration of MV. The mean duration of ventilation was 8.45 ± 4.09 days in the ET group and 13.73 ± 4.47 days in the LT group. These findings are consistent with previous investigations reporting shorter ventilatory support after earlier tracheostomy. A study evaluating patients with TBI demonstrated a significant reduction in ventilator duration in the ET group (9.63 ± 1.87 days) compared with the LT group (17.81 ± 6.66 days) [7]. Reduced MV duration after tracheostomy within 3-5 days has also been reported [8]. Additional evidence has shown fewer ventilator days among patients receiving ET compared with delayed tracheostomy (8.1 vs 11.7 days) [9]. The findings of the current study support an association between ET and shorter ventilatory duration, although this association may also be influenced by clinical selection and baseline patient differences. Duration of MV is a multifactorial outcome in severe TBI and may be influenced by neurological severity, airway reflexes, respiratory complications, associated injuries, sedation exposure, VAP, and readiness for weaning. The present study did not perform logistic regression or multivariable regression to adjust for these factors. Therefore, the shorter duration of MV observed in the ET group should be interpreted only as an unadjusted association and not as evidence that ET independently reduced ventilator duration. This substantially limits the internal validity of the primary outcome comparison.

The ET group also had a significantly shorter ICU stay than the LT group, with mean ICU stays of 11.93 ± 5.24 days and 19.00 ± 7.02 days, respectively. Transfer to the general ward occurred earlier in the ET group (11.75 ± 5.18 days) than in the LT group (17.35 ± 5.19 days). Comparable findings have been reported in neurosurgical populations, where delayed tracheostomy was associated with longer ICU stay (31.1 vs 19.9 days) [10]. Critically ill adults undergoing percutaneous tracheostomy have also shown reduced ICU stay when the procedure was performed earlier (21 vs 40.14 days) [11]. ET has further been associated with shorter ICU stay and fewer ventilator days in patients requiring prolonged ventilatory support [12]. Early intervention within three days has also been associated with significantly shorter ICU stay compared with delayed tracheostomy (14.9 ± 3.6 vs 17.2 ± 4.6 days) [13]. These findings suggest that ET may be associated with reduced intensive care burden in selected patients, although causal inference is limited by the observational nature of the study.

The prevalence of VAP was lower in the ET group (8.3%) than in the LT group (20.0%), but the difference was not statistically significant. Reduced ventilatory duration and shorter ICU stay without increased VAP risk after ET have been described previously [16]. ET has also been associated with reductions in ICU stay, hospital stay, and VAP incidence in surgical intensive care settings [17]. However, not all studies have demonstrated a significant reduction in pneumonia rates, suggesting that the relationship between tracheostomy timing and VAP may vary according to patient population, diagnostic criteria, and institutional care practices [4]. In the present study, the non-significant VAP difference should be interpreted cautiously and should not be presented as definitive evidence that ET reduces VAP.

Total hospital stay was significantly shorter in the ET group (18.63 ± 14.18 days) compared with the LT group (23.27 ± 6.40 days). Final clinical outcomes also differed significantly between groups, with clinical improvement observed in 81.7% of patients in the ET group compared with 45.0% in the LT group. Complications were more common in the LT group. Previous studies have reported reduced MV duration, shorter ICU and hospital stay, and earlier recovery from ventilator dependence with ET, without consistent differences in pneumonia occurrence [18]. The present findings are consistent with the literature suggesting that ET may be associated with better short-term clinical outcomes in severe TBI patients requiring ventilatory support.

Although the findings suggest better short-term outcomes in patients undergoing ET, they should be interpreted in the context of the retrospective observational design. Tracheostomy timing was based on clinical judgment rather than random allocation, and patients selected for ET may have differed from those managed later. The higher baseline GCS values observed in the ET group may also have contributed to better clinical outcomes. Therefore, the observed associations should not be interpreted as definitive evidence of causality.

Limitations of the study

This study has several limitations. There is no universally accepted definition of early and late tracheostomy in the available literature, and different studies have used time frames ranging from three days to more than 10 days, which limits direct comparison across studies [1]. The retrospective observational design also introduces a risk of selection bias because tracheostomy timing was determined by clinical judgment rather than randomisation or a standardised allocation protocol.

The early and late tracheostomy groups were not fully comparable in terms of available neurological severity indicators. Although all patients had severe TBI with GCS <8, the ET group had higher mean GCS values on day 1 and day 3 than the LT group. This baseline imbalance may have independently influenced ventilation duration, ICU stay, hospital stay, complications, and final clinical outcome. Although patients with severe chest or abdominal injuries and primary respiratory failure unrelated to head injury were excluded, other unmeasured confounders, including injury severity, intracranial pressure management, radiological findings, comorbidities, treatment variation, and institutional decision-making, may have influenced outcomes. Therefore, the study groups cannot be considered severity-matched, and the observed outcome differences should not be interpreted as effects of tracheostomy timing alone.

No a priori sample size calculation or power analysis was performed. The study used a retrospective census approach by including all eligible patients admitted during the predefined 18-month period; therefore, the available sample was determined by record availability rather than statistical power estimation. This limits the precision and generalisability of the findings. Continuous variables were reported as mean ± SD and compared using Student’s t-test; however, complete raw data were not uniformly available for all variables to permit robust assessment of distributional assumptions or non-parametric reanalysis. If any continuous variable had a skewed distribution, mean ± SD and Student’s t-test may not have been the most appropriate descriptive and inferential approach. Thus, the statistical comparisons should be regarded as exploratory unadjusted analyses rather than definitive inferential evidence.

The primary outcome, duration of MV, is affected by multiple dependent clinical variables, including TBI severity, associated injuries, sedation exposure, VAP, respiratory status, secretion burden, and weaning readiness. Logistic regression or multivariable regression was not performed because complete covariate data were not consistently available in the retrospective records. Therefore, the study cannot determine whether ET independently predicted shorter MV duration. This substantially limits the internal and external validity of the findings. The absence of severity adjustment or multivariable modelling is particularly important because duration of MV was the primary outcome and may have been influenced by factors other than tracheostomy timing.

Some outcomes, including clinical improvement and complications, were based on documentation in hospital records and may be subject to variability in retrospective data recording. Documentation of end-tidal carbon dioxide confirmation after tracheostomy tube placement was not uniformly available in the retrospective records. Although tube placement was documented clinically using bilateral chest expansion, auscultation, oxygen saturation, and ventilator parameters, uniform recorded capnography data were not available for all patients. This reflects incomplete retrospective documentation and variable availability of recorded end-tidal carbon dioxide data rather than absence of clinical confirmation of tube placement. Nevertheless, lack of uniform end-tidal carbon dioxide documentation remains an important procedural documentation limitation.

The study focused mainly on short-term hospital outcomes and did not assess long-term neurological recovery, functional status, quality of life, or post-discharge mortality. Because this was a single-centre study, the findings may not be fully generalisable to other centres with different clinical protocols, expertise, and available resources. Overall, the findings should be interpreted cautiously as unadjusted associations from a retrospective single-centre cohort with limited internal and external validity.

## Conclusions

The findings of this study suggest that ET, performed within four days of injury, was associated with shorter duration of MV, reduced ICU stay, earlier transfer to the general ward, and better short-term clinical outcomes compared with LT in patients with severe TBI. The ET group also showed fewer complications and a lower frequency of VAP, although the difference in VAP was not statistically significant. These findings should be interpreted as unadjusted associations rather than evidence that ET independently caused improved outcomes. The absence of prospective sample size calculation, incomplete assessment of normality assumptions, lack of severity matching, and absence of multivariable regression substantially limit the internal and external validity of the findings. Further prospective, multicentre studies using standardised timing criteria, severity-adjusted analysis, adequate sample size calculation, and long-term neurological follow-up are needed to confirm whether ET independently improves outcomes in severe TBI patients.
